# Phylogenomics of Plant NLR Immune Receptors to Identify Functionally Conserved Sequence Motifs

**DOI:** 10.21769/BioProtoc.5023

**Published:** 2024-07-05

**Authors:** Toshiyuki Sakai, AmirAli Toghani, Hiroaki Adachi

**Affiliations:** 1 Laboratory of Crop Evolution, Graduate School of Agriculture, Kyoto University, Mozume, Muko, Kyoto, Japan; 2 The Sainsbury Laboratory, University of East Anglia, Norwich Research Park, Norwich, UK; 3 JST-PRESTO, 4-1-8, Honcho, Kawaguchi, Saitama, Japan

**Keywords:** Gene annotation, Phylogenetic analysis, Motif prediction, NLR immune receptors, Plant immunity

## Abstract

In recent years, the increase in genome sequencing across diverse plant species has provided a significant advantage for phylogenomics studies, allowing the analysis of one of the most diverse gene families in plants: nucleotide-binding leucine-rich repeat receptors (NLRs). However, due to the sequence diversity of the NLR gene family, identifying key molecular features and functionally conserved sequence patterns is challenging through multiple sequence alignment. Here, we present a step-by-step protocol for a computational pipeline designed to identify evolutionarily conserved motifs in plant NLR proteins. In this protocol, we use a large-scale NLR dataset, including 1,862 NLR genes annotated from monocot and dicot species, to predict conserved sequence motifs, such as the MADA and EDVID motifs, within the coiled-coil (CC)-NLR subfamily. Our pipeline can be applied to identify molecular signatures that have remained conserved in the gene family over evolutionary time across plant species.

Key features

• Phylogenomics analysis of plant NLR immune receptor family.

• Identification of functionally conserved sequence patterns among plant NLRs.

## Background

Nucleotide-binding leucine-rich repeat receptors (NLRs) play a pivotal role in plants' innate immune system by recognizing pathogen-secreted molecules, ultimately triggering robust defense responses known as the hypersensitive response [1]. Plant NLRs are characterized by three principal domains: an N-terminal coiled coil (CC) or toll/Interleukin-1 receptor (TIR) domain, a central nucleotide-binding domain with APAF-1, various R proteins, and a CED-4 (NB-ARC) domain, and a C-terminal leucine-rich repeat (LRR) domain [2]. Although plant NLR proteins share the common domain architecture, plant NLRs often undergo rapid evolution and remarkable sequence diversification, reflecting a continuous arms race between plants and pathogens [3–5]. This sequence diversity in the NLR immune receptor family enables plants to recognize a wide range of pathogen-derived molecules, which are typically fast-evolving, to modulate the multi-layered plant immune system for successful pathogen infection. Therefore, understanding the evolutionary dynamics of the NLR gene family across diverse plant species provides valuable insights into the molecular mechanisms underlying the plant immune system and also for the breeding program of disease-resistant crops.

**Figure 1.**
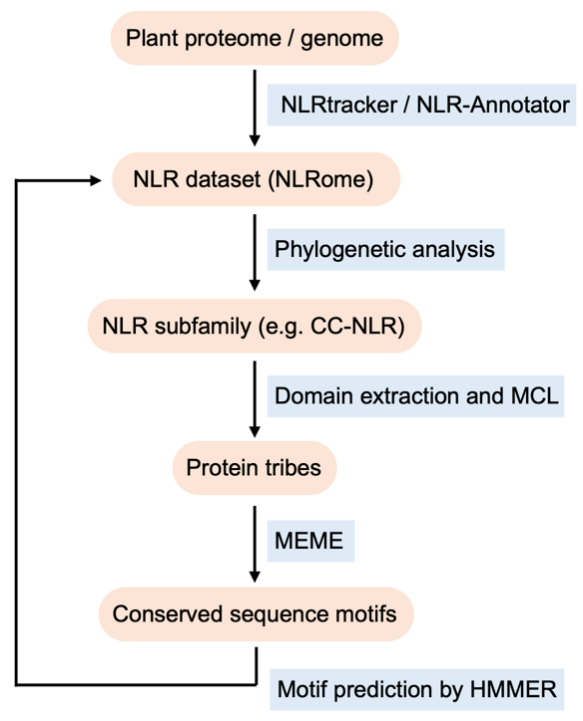
Workflow to identify conserved sequence patterns in nucleotide-binding leucine-rich repeat receptors (NLR) family proteins by phylogenomics

Previous studies have elucidated the molecular function of NLR proteins by site-directed mutations targeting conserved regions and motifs. For instance, mutations in the P-loop and MHD motifs within the central NB-ARC domain can render plant NLRs nonfunctional and autoactive, respectively [6,7]. Therefore, identifying evolutionally conserved motifs is a key strategy to pinpoint and understand yet unknown plant NLR functions. For smaller sets of sequences, multiple sequence alignment is an effective tool to uncover specific conserved regions and amino acid residues [8]. However, the multiple sequence alignment method encounters technical challenges with larger datasets, where identifying conserved regions becomes complicated due to the presence of gaps and deletions, especially in the diverse gene family of plant NLRs. To address this, we have developed a computational pipeline tailored for identifying conserved sequence patterns in the plant NLR family [9]. This approach integrates NLR gene annotation, phylogenetic analysis, clustering protein families, and prediction of conserved sequence motifs. Here, using a test NLR dataset from six representative plant species (previously analyzed in Adachi et al. [9]), we provide a step-by-step procedure to identify functionally conserved motifs in CC-type NLR (CC-NLR) subfamily (Figure 1). Given the development of genome sequencing technologies and rising access to genome databases of diverse plant species, our pipeline has the potential to be applied for further phylogenomics studies of plant NLR and other gene families.

## Equipment

1. 64-bit Linux operating system; alternatively, Mac OS X operating system

## Software and datasets

1. Protein or nucleotide sequences from reference genome databases
2. InterProScan 5.53-87.0 [10]
3. NLRtracker v1.0.3 [2]
4. NLR-Annotator v2.1 [11]
5. MAFFT v7 [12]
6. RAxML v8.2.12 [13]
7. iTOL (Interactive Tree Of Life) [14]
8. BLAST+ v2.12.0 [15]
9. MCL v14-137 [16]
10. MEME Suite v5.5.5 [17]; same analysis can be performed through MEME website (https://meme-suite.org/meme/tools/meme)
11. HMMER v3.4 [18]
12. Test data are included as supplemental datasets in this manuscript and have been deposited in GitHub: https://github.com/slt666666/MADA_motif_protocol. Supplemental Python scripts can be used through Google Collaboratory: https://colab.research.google.com/github/slt666666/MADA_motif_protocol/blob/master/Supplemental_scripts.ipynb (Access date, 22/01/24). The instructions for using Supplemental Scripts are provided in Supplemental video file (Video S1).

## Procedure

- **Software installation**
  1. InterProScan 5.53-87.0
    InterProScan is a software that characterizes protein function. This program can be downloaded and installed by following the instructions provided at https://www.ebi.ac.uk/interpro/download/. It is compatible with 64-bit Linux operating systems. In this protocol, the InterProScan is utilized in the NLRtracker pipeline.
  2. NLRtracker v1.0.3
    NLRtracker is an NLR annotation tool that utilizes a protein sequence file as the input dataset. This program can be downloaded and installed by following the instructions provided at https://github.com/slt666666/NLRtracker. NLRtracker is more sensitive and accurate than other available tools for extracting NLRs from a given plant proteome [2]. Alternatively, NLR-Annotator v2.1 can be downloaded and installed by following the instructions available at https://github.com/steuernb/NLR-Annotator. NLR-Annotator is an NLR annotation tool designed to work with nucleotide sequence files as input datasets. This program is suitable for users who do not have access to a Linux system.
  3. MAFFT v7
    MAFFT is a program for multiple sequence alignment. This program can be downloaded and installed by following the instructions provided at https://mafft.cbrc.jp/alignment/software/.
  4. RAxML v8.2.12
    RAxML is a program for maximum likelihood-based inference of large phylogenetic trees. To download and install this program, please refer to PART and in the manual available at https://cme.h-its.org/exelixis/resource/download/NewManual.pdf. Alternatively, RAxML-NG v1.2.1 can be downloaded and installed on Unix/Linux and macOS systems by following the instructions provided at https://github.com/amkozlov/raxml-ng?tab=readme-ov-file.
  5. BLAST+ v2.12.0
    BLAST+ is a command-line tool to run BLAST in your own local environment. This program can be downloaded and installed by following the instructions provided at https://www.ncbi.nlm.nih.gov/books/NBK569861/.
  6. MCL v14-137
    MCL software is a program for clustering weighted or simple networks. To install blast packages in the MCL software, “--enable-blast” option is required. The installation commands are as follows:
    wget http://www.micans.org/mcl/src/mcl-14-137.tar.gz
    tar -zxvf mcl-14-137.tar.gz
    cd mcl-14-137
    ./configure --enable-blast
    Make
    make install
  7. MEME Suite v5.5.5
    MEME Suite is a motif-based sequence analysis tool. This tool can be downloaded and installed by following the instructions provided at https://meme-suite.org/meme/doc/install.html. Alternatively, the same analysis can be performed through the MEME website at https://meme-suite.org/meme/tools/meme.
  8. HMMER v3.4
    HMMER is utilized for searching sequence homologs from sequence databases and for making sequence alignments. This program can be installed by following the instructions provided at https://github.com/EddyRivasLab/hmmer.
- **Annotate NLR gene family from proteome datasets**
  1. Download protein sequence files from reference genome databases.
    As a test dataset, we used proteomes from six representative plant species: *Arabidopsis thaliana, Beta vulgaris* (sugar beet), *Solanum lycopersicum* (tomato), *Nicotiana benthamiana, Oryza sativa* (rice), and *Hordeum vulgare* (barley). The protein sequences were downloaded from the reference genome databases of *Arabidopsis* (https://www.araport.org/, Araport11), sugar beet (http://bvseq.molgen.mpg.de/index.shtml, RefBeet-1.2), tomato (https://solgenomics.net/, tomato ITAG release 2.4), *N. benthamiana* (https://solgenomics.net/, *N. benthamiana* genome v0.4.4), rice (http://rice.plantbiology.msu.edu/, rice gene models in Release 7) and barley (https://www.barleygenome.org.uk/, IBSC_v2) as used in Adachi et al. [9]. The protein sequences were compiled into a single fasta file named “NLRtracker_input_protein.fasta” (Dataset S1).
  2. Annotate NLRs from protein sequences.
    NLRs were annotated from the **input** protein sequence file **“NLRtracker_input_protein.fasta”** by running NLRtracker using the following command:
    ./NLRtracker -s NLRtracker_input_protein.fasta -o NLRtracker_output
    The **output** NLR protein sequences were saved as **“NLR.fasta”** in the “NLRtracker_output” folder. In total, we identified 1,862 NLRs from six representative plant species.
*Note: In a previous study, we used a tool, NLR-Annotator, to annotate NLR genes [11]. However, since NLR-Annotator may not detect a few functionally validated NLRs (e.g., ADR1), we employed NLRtracker [2] in this protocol. Therefore, test datasets in the following analyses slightly differ from the data reported in Adachi et al. [9].*
  3. Extract specific NLR subfamily sequences based on phylogenetic analysis.
    In a previous study [9], we characterized a conserved sequence pattern (MADA motif) crucial for CC-NLRs to trigger immune responses. To identify conserved sequence patterns in each NLR subfamily, we initially classified NLRs through phylogenetic analysis. Here, the NLR sequences obtained in step B2 were combined with 31 functionally characterized CC-NLRs and saved as “NLR_set.fasta” (Dataset S2). Protein sequences in the **input** file **“NLR_set.fasta”** were aligned using MAFFT:
    mafft NLR_set.fasta > NLR_set_alignment_output.fasta
    For the phylogenetic analysis of the NLR family, NB-ARC domain sequences were extracted from the **output** alignment file **“NLR_set_alignment_output.fasta”** based on the NB-ARC domain sequence of *Arabidopsis* ZAR1 (Dataset S3). Extraction of NB-ARC domain sequences can be performed manually using alignment software or using our script (Supplemental script 1). In this script, protein sequences lacking the intact p-loop motif (G/AxxxxGKT/S) required for NLR protein function are automatically discarded from the dataset. Sequence gaps in the aligned NB-ARC domain sequences are also automatically deleted in this script. The sequences were saved as **“NLR_set_alignment_NBARC_RemGap.fasta”** (Dataset S4), which can be used as the **input** file for further phylogenetic analysis.
*Note: We use conserved NB-ARC domain sequences for phylogenetic analyses of the NLR gene family because other domains, such as N-terminal domains and C-terminal LRR domain, are often too diversified and not suitable for inferring phylogenetic relations in NLRs.*
    The maximum likelihood phylogenetic tree was inferred by RAxML using the following command:
    raxmlHPC-PTHREADS-AVX2 -s NLR_set_alignment_NBARC_RemGap.fasta -n NLR_MLtree -m PROTGAMMAAUTO -f a -# 100 -x 1024 -p 121
*Note: The ‘-f’ and ‘-#’ options were set for 100 iterations of bootstrap. The ‘-x’ and ‘-p’ options were random seeds.*
    NLRs that belong to the CC-NLR phylogenetic subclade were classified with functionally characterized CC-NLRs on the NLR phylogenetic tree **output** file **“RAxML_bipartitions.NLR_MLtree”** (Figure 2; Dataset S5). We extracted 1,305 protein IDs (Dataset S6) of the CC-NLR clade in the NLR phylogenetic tree using iTOL [14]. For further sequence analysis, we extracted protein sequences of CC-NLRs from the **input** file **“NLR_set.fasta”** (Dataset S2) and **“CCNLR_IDs.txt”** (Dataset S6) using Supplemental script 2 and saved them as the **output** file **“CCNLR_set.fasta”** (Dataset S7).
    **Figure 2.**
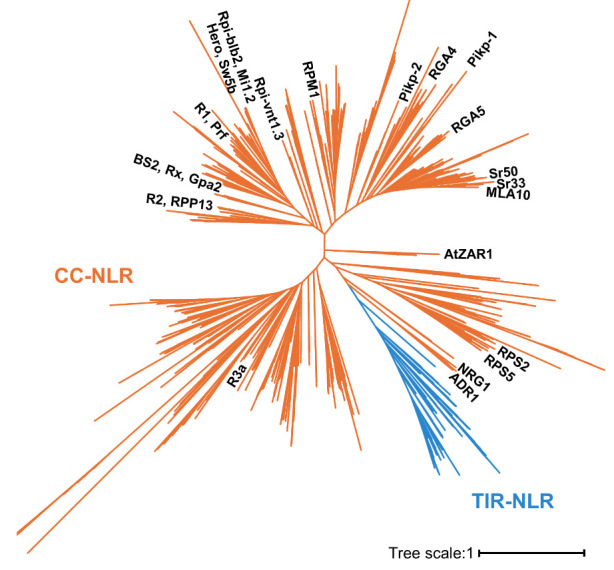
Maximum likelihood phylogenetic tree for the classification of nucleotide-binding leucine-rich repeat receptors (NLR) subfamilies. Functionally characterized CC-NLRs are labeled in the phylogenetic tree using iTOL (Interactive Tree Of Life) [14]. The orange branches represent coiled-coil (CC)-NLRs used for further sequence analyses.
- **Classify N-terminal domain sequences of CC-NLRs using Markov cluster (MCL) algorithm**
  1. Extract N-terminal sequences from CC-NLRs.
    Sequences prior to the NB-ARC domain were defined as N-terminal domain sequences. To extract N-terminal domain sequences, protein sequences in the **input** file **“CCNLR_set.fasta”** were aligned using MAFFT as described in section B3. N-terminal domain sequences of CC-NLRs were extracted from the alignment result, based on the start position of the NB-ARC domain of *Arabidopsis* ZAR1 (Dataset S3). Extraction of the N-terminal domain sequences can be done manually using alignment software or using our script (Supplemental script 3). The extracted N-terminal domain sequences were saved as the **output** file **“CCNLR_Ndomain_set.fasta”** (Dataset S8).
  2. Cluster N-terminal domain sequences into protein families.
    N-terminal domain sequences of CC-NLRs were clustered into protein families based on sequence similarity using the MCL algorithm. The method is based on a graph that contains similarity information obtained from BLAST searches. The similarity information was obtained using the following commands with the **input** file **“CCNLR_Ndomain_set.fasta”** (Dataset S8):
    makeblastdb -in CCNLR_Ndomain_set.fasta -dbtype prot
    blastp -query CCNLR_Ndomain_set.fasta -db CCNLR_Ndomain_set.fasta -out blast_results.txt -evalue 1e-8
*Note: The outcome of this BLAST search is dependent on the e-value cutoff set by investigators. We set the BLASTP e-value cutoff to <10^−8^ by checking the sequence similarity of raw BLAST hits.*
    Clustering N-terminal domain sequences from the BLAST output file was performed using the following mclblastline command:
    mclblastline --mcl-I=1.4 blast_results.txt
*Note: The mcl-I option, which affects cluster granularity, was set to 1.4.*
    Provided N-terminal domain sequences were classified into several tribes in the **output** file **“dump.out.blast_results.txt.l14”** (Dataset S9). Among the output tribes, we focused on a tribe including ZAR1, RPP13, R2, and Rpi-vnt1.3 (tribe 3) for further sequence analyses, as described in Adachi et al. [9]. We then extracted IDs of N-terminal domain sequences from CC-NLRs grouped into tribe 3 using Supplemental script 4. For the analysis of conserved sequences, we extracted N-terminal domain sequences of tribe 3 from the **input** file **“CCNLR_Ndomain_set.fasta”** (Dataset S8) using Supplemental script 2 and saved as fasta file **“Nseq_Tribe3.fasta”** (Dataset S10).
- **Identify conserved sequence motifs in the N-terminal domain of NLRs**
  1. Identify conserved sequence patterns in the N-terminal domain tribes.
    Conserved sequence patterns in tribe 3 were identified using MEME (Multiple EM for Motif Elicitation) with the following command with the **input** file **“Nseq_Tribe3.fasta”** (Dataset S10):
    meme Nseq_Tribe3.fasta -protein -oc. -nostatus -time 14400 -mod zoops -nmotifs 5 -minw 6 -maxw 50 -objfun classic -minsites 62 -markov_order 0
*Note: The ‘-minsites’ cutoff was set to 70% of the number of sequences to identify high occurrent sequence motifs. Other options are default settings in the MEME website (* *https://meme-suite.org/meme/tools/meme* ).
    From our test data, we identified five conserved sequence patterns in the N-terminal domain of tribe 3 CC-NLRs (Figure 3). Among the identified motifs in the **output “meme.html”**, a motif located at the very N terminus was defined as the MADA motif based on the deduced 21 amino acid consensus sequence “MADAxVSFxVxKLxxLLxxEx” [9], conserved in approximately 78% of tribe 3 CC-NLRs. The EDVID motif, which functions in stabilizing the structure of CC-NLR proteins [19], is conserved in approximately 85% of tribe 3 CC-NLRs (Figure 3).
    **Figure 3.**
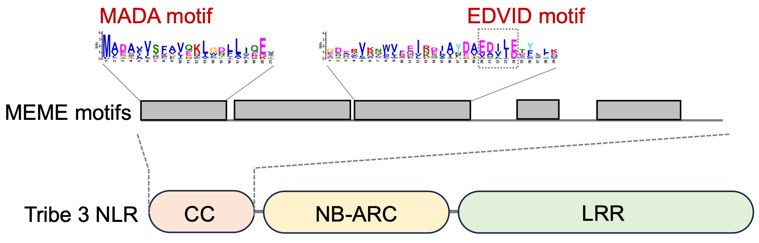
Consensus sequence patterns detected in the N-terminal domain of tribe 3 coiled-coil nucleotide-binding leucine-rich repeat receptors (CC-NLRs). Conserved motifs were identified by MEME from 88 tribe 3 CC-NLR members. The motif logos describe the N-terminal consensus patterns, as reported in Adachi et al. [9]. Figure is modified from Adachi et al. [9].
- **Search the MADA motif using the hidden Markov model (HMM)**
  1. Build the HMM of the conserved sequence pattern corresponding to the MADA motif.
    Protein sequences corresponding to the MADA motif were extracted from a MEME output file and saved as the **input** file **“MEME_alignment.txt”** (Dataset S11). The HMM for the MADA motif was built using HMMER with the following command:
    hmmbuild MADA.hmm MEME_alignment.txt
  2. Perform HMMER search to query NLR proteomes using the MADA motif HMM.
    HMMER detects sequence homologs and calculates HMM scores based on the degree of match. We performed a HMMER search to query NLR proteomes (Dataset S2) using the MADA motif HMM with the following command:
    hmmsearch --max --tblout MADA.hmmsearch.tsv -o MADA.hmmsearch.txt MADA.hmm NLR_set.fasta
    We set an HMM score cutoff at 10.0, which is most optimal for high-confidence searches of MADA containing CC-NLR proteins (MADA-CC-NLRs) [9]. We also defined NLR proteins with HMM scores from 0 to 10.0 as MADA-like CC-NLRs. From our test data, we identified 108 MADA-CC-NLRs and 161 MADA-like CC-NLRs. Based on conserved sequence patterns of NLRs, we can predict evolutionally conserved molecular functions of NLRs and can apply this for mutant analyses in molecular biology, biochemistry, and cell biology experiments as described in recent studies [1,9,20,21].

## Validation of protocol

This protocol or parts of it has been used and validated in the following research article(s):

- Adachi et al. [9]. An N-terminal motif in NLR immune receptors is functionally conserved across distantly related plant species. eLife (Figures 3–6).
- Chia et al. [22]. The N-terminal domains of NLR immune receptors exhibit structural and functional similarities across divergent plant lineages. Plant Cell (Figures 3 and 5).

## Supplementary information

The following supporting information can be downloaded here:

1. Dataset S1. Proteome fasta file used as input for NLRtracker.
2. Dataset S2. Test dataset of full-length NLR proteins used for phylogenetic analysis.
3. Dataset S3. NB-ARC domain sequence of Arabidopsis ZAR1.
4. Dataset S4. Test dataset of aligned NB-ARC domain sequences used for phylogenetic analysis.
5. Dataset S5. Test dataset of maximum likelihood tree.
6. Dataset S6. Test dataset of protein IDs of CC-NLR subfamily.
7. Dataset S7. Test dataset of CC-NLR subfamily sequences.
8. Dataset S8. Test dataset of N-terminal domain sequences extracted from CC-NLRs.
9. Dataset S9. Test dataset of protein IDs for each tribe.
10. Dataset S10. Test dataset of N-terminal domain sequences extracted from tribe 3.
11. Dataset S11. Test dataset of the MADA motif sequences extracted in MEME.
12. Video S1. Simple instructions for using Supplemental Scripts.

## References

[1] Contreras M. P., Lüdke D., Pai H., Toghani A. and Kamoun S. (2023). NLR receptors in plant immunity: making sense of the alphabet soup. EMBO Rep. 24(10): e57495. 37602936 10.15252/embr.202357495 PMC10561179

[2] Kourelis J., Sakai T., Adachi H. and Kamoun S. (2021). RefPlantNLR is a comprehensive collection of experimentally validated plant disease resistance proteins from the NLR family. PLoS Biol. 19(10): e3001124. 34669691 10.1371/journal.pbio.3001124 PMC8559963

[3] Van de Weyer A. L., Monteiro F., Furzer O. J., Nishimura M. T., Cevik V., Witek K., Jones J. D., Dangl J. L., Weigel D., Bemm F., .(2019). A Species-Wide Inventory of NLR Genes and Alleles in *Arabidopsis thaliana* . Cell. 178(5): 1260 1272 1272 .e14. 31442410 10.1016/j.cell.2019.07.038 PMC6709784

[4] Lee R. R. and Chae E. (2020). Variation Patterns of NLR Clusters in *Arabidopsis thaliana* Genomes. Plant Commun. 1(4): 100089. 33367252 10.1016/j.xplc.2020.100089 PMC7747988

[5] Prigozhin D. M. and Krasileva K. V. (2021). Analysis of intraspecies diversity reveals a subset of highly variable plant immune receptors and predicts their binding sites. Plant Cell. 33(4): 998-1015. 33561286 10.1093/plcell/koab013 PMC8226289

[6] Bendahmane A., Farnham G., Moffett P. and Baulcombe D. C. (2002). Constitutive gain‐of‐function mutants in a nucleotide binding site–leucine rich repeat protein encoded at the *Rx* locus of potato. Plant J. 32(2): 195-204. 12383085 10.1046/j.1365-313x.2002.01413.x

[7] Tameling W. I. L., Elzinga S. D. J., Darmin P. S., Vossen J. H., Takken F. L. W., Haring M. A. and Cornelissen B. J. C. (2002). The Tomato *R* Gene Products I-2 and Mi-1 Are Functional ATP Binding Proteins with ATPase Activity. Plant Cell. 14(11): 2929-2939. 12417711 10.1105/tpc.005793 PMC152737

[8] Contreras M. P., Pai H., Selvaraj M., Toghani A., Lawson D. M., Tumtas Y., Duggan C., Yuen E. L. H., Stevenson C. E. M., Harant A., .(2023). Resurrection of plant disease resistance proteins via helper NLR bioengineering. Sci Adv. 9(18): eadg3861. 37134163 10.1126/sciadv.adg3861 PMC10156107

[9] Adachi H., Contreras M. P., Harant A., Wu C. h., Derevnina L., Sakai T., Duggan C., Moratto E., Bozkurt T. O., Maqbool A., .(2019). An N-terminal motif in NLR immune receptors is functionally conserved across distantly related plant species. eLife. 8: e49956. 31774397 10.7554/eLife.49956 PMC6944444

[10] Jones P., Binns D., Chang H. Y., Fraser M., Li W., McAnulla C., McWilliam H., Maslen J., Mitchell A., Nuka G., .(2014). InterProScan 5: genome-scale protein function classification. Bioinformatics 30(9): 1236-1240. 24451626 10.1093/bioinformatics/btu031 PMC3998142

[11] Steuernagel B., Witek K., Krattinger S. G., Ramirez-Gonzalez R. H., Schoonbeek H. j., Yu G., Baggs E., Witek A. I., Yadav I., Krasileva K. V., .(2020). The NLR-Annotator Tool Enables Annotation of the Intracellular Immune Receptor Repertoire. Plant Physiol. 183(2): 468-482. 32184345 10.1104/pp.19.01273 PMC7271791

[12] Katoh K. and Standley D. M. (2013). MAFFT Multiple Sequence Alignment Software Version 7: Improvements in Performance and Usability. Mol Biol Evol. 30(4): 772-780. 23329690 10.1093/molbev/mst010 PMC3603318

[13] Stamatakis A. (2014). RAxML version 8: a tool for phylogenetic analysis and post-analysis of large phylogenies. Bioinformatics 30(9): 1312-1313. 24451623 10.1093/bioinformatics/btu033 PMC3998144

[14] Letunic I. and Bork P. (2021). Interactive Tree Of Life(iTOL) v5: an online tool for phylogenetic tree display and annotation. Nucleic Acids Res. 49: W293-W296. 33885785 10.1093/nar/gkab301 PMC8265157

[15] Camacho C., Coulouris G., Avagyan V., Ma N., Papadopoulos J., Bealer K. and Madden T. L. (2009). BLAST+: architecture and applications. BMC Bioinf. 10(1): 421. 10.1186/1471-2105-10-421 PMC2803857 20003500

[16] Van Dongen S. (2008). Graph Clustering Via a Discrete Uncoupling Process. SIAM J Matrix Anal Appl. 30(1): 121-141.

[17] Bailey T. L., Johnson J., Grant C. E. and Noble W. S. (2015). The MEME Suite. Nucleic Acids Res. 43: W39-W49. 25953851 10.1093/nar/gkv416 PMC4489269

[18] Eddy S. R. (1998). Profile hidden Markov models. Bioinformatics 14(9): 755-763. 9918945 10.1093/bioinformatics/14.9.755

[19] Förderer A., Li E., Lawson A. W., Deng Y. n., Sun Y., Logemann E., Zhang X., Wen J., Han Z., Chang J., .(2022). A wheat resistosome defines common principles of immune receptor channels. Nature. 610(7932): 532-539. 36163289 10.1038/s41586-022-05231-w PMC9581773

[20] Duggan C., Moratto E., Savage Z., Hamilton E., Adachi H., Wu C. H., Leary A. Y., Tumtas Y., Rothery S. M., Maqbool A., .(2021). Dynamic localization of a helper NLR at the plant–pathogen interface underpins pathogen recognition. Proc Natl Acad Sci USA. 118(34): e2104997118. 34417294 10.1073/pnas.2104997118 PMC8403872

[21] Ahn H., Lin X., Olave‐Achury A. C., Derevnina L., Contreras M. P., Kourelis J., Wu C., Kamoun S. and Jones J. D. G. (2023). Effector‐dependent activation and oligomerization of plant NRC class helper NLRs by sensor NLR immune receptors Rpi‐amr3 and Rpi‐amr1. EMBO J. 42(5): e111484. 36592032 10.15252/embj.2022111484 PMC9975942

[22] Chia K. S., Kourelis J., Teulet A., Vickers M., Sakai T., Walker J. F., Schornack S., Kamoun S. and Carella P. (2024). The N-terminal domains of NLR immune receptors exhibit structural and functional similarities across divergent plant lineages. Plant Cell koae113. Advance online publication. 10.1093/plcell/koae113 PMC11218826 38598645

